# Curcumin effects on myeloperoxidase, interleukin-18 and matrix metalloproteinase-9 inflammatory biomarkers in patients with unstable angina: A randomized clinical trial

**Published:** 2019

**Authors:** Amir hooshang Mohammad pour, Mostafa Dastani, Roshanak Salari, Sohrab Radbin, Soghra Mehri, Maryam Ghorbani, Asieh Karimani, Masoumeh Salari

**Affiliations:** 1 *Department of Clinical Pharmacy, School of Pharmacy, Mashhad University of Medical Sciences, Mashhad, Iran.*; 2 *Pharmaceutical Research Center, Institute of Pharmaceutical Technology, Mashhad University of Medical Sciences, Mashhad, Iran.*; 3 *Department of Cardiology, Ghaem Hospital, School of Medicine, Mashhad University of Medical Sciences, Mashhad, Iran.*; 4 *Department of Clinical Persian Pharmacy, School of Persian and Complementary Medicine, Mashhad University of Medical Sciences, Mashhad, Iran.*; 5 *Department of Pharmacodynamics and Toxicology, School of Pharmacy, Mashhad University of Medical Sciences, Mashhad, Iran.*; 6 *Department of Pharmacology and Toxicology, School of Pharmacy, Baqiyatallah University of Medical Sciences, Tehran, Iran.*; 7 *Department of Internal Medicine, Ghaem Hospital, School of Medicine, Mashhad University of Medical Sciences, Mashhad, Iran.*; † * Equal first author*

**Keywords:** Curcumin, Interlukein-18 Myeloperoxidase, Matrix metalloproteinase-9, Unstable angina

## Abstract

**Objective:**

Inflammation along with oxidative stress plays an important role in the development, progression, instability and rupture of coronary atherosclerotic plaques. Several studies introduced curcumin (diferuloylmethane) as a wonderful chemical in *Curcuma longa* (turmeric) with appropriate anti-inflammatory and antioxidant effects. The effect of curcumin on inflammatory biomarkers was assessed in several clinical trials. This study was designed to evaluate the effect of curcumin on three pro-inflammatory biomarkers in patients with unstable angina.

**Materials and Methods:**

Forty patients with unstable angina who met the inclusion criteria, participated in this double-blind randomized clinical trial. Patients were randomly divided into two groups. The patients in the treatment group received nanocurcumin 80 mg per day for 5 days and the control group received placebo 80 mg per day for five days. Blood samples were obtained before the administration, and also 1, 2 and 4 days after taking the treatment. Serum concentrations of Myeloperoxidase (MPO), matrix metalloproteinase-9 (MMP-9) and interleukin 18 (IL-18) biomarkers were measured by ELISA.

**Results:**

There was no significant difference in concentration of these biomarkers before the administration and 1, 2 and 4 days after the start of the trial, between the two groups; however, the concentration of IL-18 on the first day significantly varied between the groups.

**Conclusion:**

Based on the findings of this study, administration of nanocurcumin capsules at the dose of 80 mg per day for 5 days, did not significantly decrease inflammatory biomarkers in patients with unstable angina.

## Introduction

Inflammation is a major factor in the initiation and progression of various types of serious diseases such as arthritis, cardiovascular, neurodegenerative, metabolic diseases and different types of cancer (Libby Peter et al., 2002 ▶; Lu et al., 2006 ▶; Lucas et al., 2006 ▶; Sokolove and Lepus, 2013 ▶). The role of inflammation has been well studied specially in atherosclerosis and it was introduced as an important and critical pathological state that leads to serious cardiovascular diseases such as coronary artery disease (CAD). All stages in the course of initiation to progression and instability of atheroma are worsened by inflammatory responses (Libby, 2000 ▶). The vascular endothelium could be damaged by various harmful factors including trauma, oxidative stress or modified lipoproteins (Alique et al., 2015 ▶). Adhesion molecules, such as intercellular adhesion molecule 1 (ICAM-1), vascular adhesion molecule 1 (VCAM-1) and selectins which are all produced by injured endothelium, bonds leukocytes leading to endothelial dysfunction (Čejková et al., 2016 ▶). Following adhesion and migration of monocytes, they differentiate into tissue macrophages (Čejková et al., 2016 ▶). The uptake of low-density lipoproteins (LDLs) by macrophages transform them into the lipid-laden foam cells (Zakynthinos and Pappa, 2009 ▶). Release of inflammatory cytokines such as interleukin 1 (IL-1) and IL-6 from mononuclear cells attracts more inflammatory cells which eventually leads to higher extent of oxidative modification of LDLs. This is the way via which an atheroma could be produced (Sprague and Khalil, 2009 ▶). In fact, inflammatory signals play an important role in the development of such a lesion (Libby et al., 2002 ▶). Several subsequent events happen to manage the condition. Collagen construction activates macrophages to secrete matrix metalloproteinases (MMPs), which contain collagenase that makes the plaque ready to rupture. However, this process may cause further inflammatory reactions, platelet activation, coagulation cascade induction, and vasomotor dysfunction (Hiller et al., 2000 ▶). It was established that elevated serum levels of MMP-1, MMP-2, and MMP-9 are all correlated with acute coronary syndrome. Similar studies showed higher risk of cardiovascular death in patients with increased MMP-9 levels (Blankenberg et al., 2003 ▶).

The above-mentioned evidence have verified that accumulation of selective inflammatory mediators and oxidative stress play a key role in the initiation and progression of cardiovascular diseases. Some of the main biomarkers that are elevated in this condition, are C-reactive protein, inflammatory cytokines like IL-1, IL-6, IL-10, tumor necrosis factor alpha (TNF-α), and monocyte chemoattractant protein 1(MCP-1), cell adhesion molecules (CAMs), myeloperoxidase and MMPs (Zakynthinos and Pappa, 2009 ▶). Myeloperoxidase (MPO), a heme protein, is involved in LDL oxidation. This agent is released by degraded neutrophils, monocytes, and tissue macrophages following inflammatory events. There is an association between higher serum MPO levels and increased risk of CAD (Baldus et al., 2003 ▶). Moreover, IL-18, a proinflammatory cytokine which has an important role in the inflammatory cascade, may be related to atherosclerotic plaque progression and also increased risk of CAD (Mallat et al., 2001 ▶).

Intracellular oxidative stress occurs following various conditions such as hypertension, diabetes, hyperlipidemia and vascular hemodynamic stresses via unknown mechanism(s) (Foncea et al., 2000 ▶).

Curcumin (diferuloylmethane), a major active component of *Curcuma longa*. In ancient remedies, it is used to treat infections, wounds, bites, burns, acne and various skin conditions as well as digestive disorders such as dyspepsia (Hatcher et al., 2008 ▶). A wide range of mechanisms has been shown to mediate curcumin effects. It was revealed that curcumin has antioxidant, anti-inflammatory, antiproliferative, pro-apoptotic, antibacterial, antifungal, and antiviral effects (Aggarwal et al., 2007 ▶).

It was reported that different kinds of disorders such as neoplastic and chronic inflammatory diseases can be treated, at least in part, by curcumin administration. In this context, curcumin was used for treatment of disorders such as multiple sclerosis, cerebral injury, cardiovascular disease, asthma, bronchitis, colitis, and rheumatoid arthritis (Aggarwal and Harikumar, 2009 ▶). Considering its anti-inflammatory properties, curcumin is able to reduce the expression of enzymes such as cyclooxygenase (COX) 2 and 5 lipoxygenase, and cytokines including tumor necrosis factor (TNF), IL-1, and IL-6 via down regulation of nuclear factor kB (Zhou et al., 2011 ▶). Considering the beneficial effects of curcumin on various factors involved in atheroma formation, in this study, the effect of this active material on patients with unstable angina was investigated. 

## Materials and Methods

**Study design**

This randomized, double-blind, clinical trial was conducted at the Cardiology ward, Ghaem academic hospital, Mashhad University of Medical Sciences, Mashhad, Iran (September 2014 -May 2015). The research population was comprised of patients referring to the cardiology clinic of Ghaem Hospital who were diagnosed with unstable angina by a cardiologist, based on New York Heart Association (NYHA) 2013 guideline. The study protocol was approved by the Ethics Committee of Mashhad University of Medical Sciences. This study was registered in the Iranian Registry of Clinical Trial (IRCT No. 2013102315122N1). 

**Eligibility criteria**

Patients accepted to participate in the study by signing an informed consent form. Patients above the age of 20 years, diagnosed with unstable angina by an experienced cardiologist, were included.

Patients with renal or hepatic dysfunction, chronic inflammatory disease, history of cardiac arrhythmia, heart failure, and those who required PCI (percutaneous coronary intervention) emergency or were allergic to curcumin, pregnant or lactating women, and the patients who were receiving immunosuppressants or anti-inflammatory drugs, were all excluded.

Before therapeutic intervention, a questionnaire containing demographic data, underlying diseases, and history of any particular disease, family history, cardiovascular risk factors and medications were completed. At first, all the patients were examined by an internist and evaluated for inclusion and exclusion criteria.

Overall, out of the 64 patients with unstable angina referring to the cardiology department, 40 subjects met the criteria for enrollment. Others were excluded because of various reasons including being sensitive to curcumin (2 subjects), having liver disease (3 subjects), or kidney disease (6 subjects), refusing to take part in the experiment (6 subjects), having infectious disease (3 subjects), and receiving anti-inflammatory drugs (4 subjects). 

**Intervention**

Patients were randomly allocated into two groups by simple randomization based on computer-generated random numbers. Subjects and investigators did not know to which group a subject will be allocated. Both the assessor and the statistical analyst were blinded to the treatment allocation. Eventually, 40 patients were randomly divided into two groups. One group (A) (n=20) was administered with soft gelatin capsules containing nanocurcumin with the brand name of Sina Curcumin ^TM^, one per day for five days. Each capsule of Sina Curcumin contains 80 mg curcuminoid as nanomicelles, manufactured by Sina Daroo Company. Whereas the control group (B) (n=20) received placebo containing lactulose and starch. Placebo was administered in the same pattern as curcumin. In addition, conventional drug therapy for treatment of unstable angina (i.e. nitrates, beta blockers, angiotensin inhibitors, statin, aspirin and clopidogrel bisulfate) was administered similarly to both groups.

**Blood sampling**

Blood samples (5 ml) were obtained from patients on day 0 (before intervention), and 1, 2 and 4 after curcumin or placebo administration. The samples were introduced to sterile test tubes containing EDTA. They were centrifuged at 400 RPM for 15 minutes; then, the sera were isolated and transferred to a -70°C freezer. The serum concentration of biomarkers (MPO, IL-18 and MMP-9) was determined by an enzyme-linked immunosorbent assay (ELISA) kit (Axis-shield). 

**Statistical analysis**

The data were analyzed using SPSS version 16 software. Frequency was used to describe the discrete variables and the mean and standard error of mean (SEM) were used for description of continuous variables. Normality of data distribution was tested by Kolmogorov-Smirnov test. For normal and abnormal data, parametric and non-parametric tests were used, respectively. To investigate the relationship among different parameters, the crosstabs test; also, in order to comparing the results between two groups, independent sample T-test and Mann Whitney tests were used. The significance level in all tests, was considered p<0.05.

## Results

**Demographic data**

The mean age of the study population was 61.31 years. We also considered other risk factors, such as age, hypertension, hypercholesterolemia, smoking and diabetes. There was no significant difference in various parameters between curcumin and placebo groups (Table 1).

**Table 1:** Demographic characteristics of curcumin and placebo groups

Risk factor	Curcumin group	Placebo
Number	20	20
Gender (Male)%	41.2	42.9
Smoking or addiction (%)	31.3	25
Hypertension (%)	68.8	62.4
Hyperlipidemia (%)	31.2	50
Diabetes (%)	25	37.6
Age (year)	59.7±10.5	61.3±11.4

**Comparison of the biomarkers concentrations between the two groups**

**1. Myeloperoxidase (MPO) Concentration**

**Figure 1:**
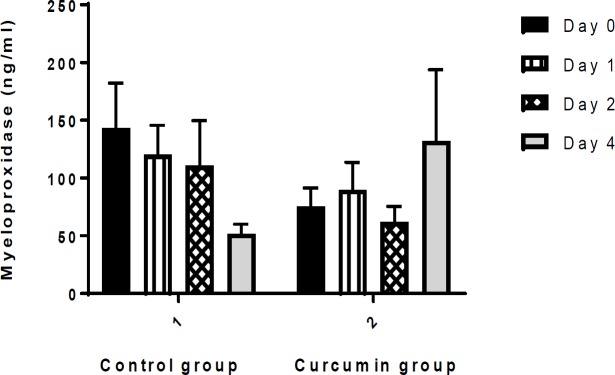
Comparison of myeloperoxidase (MPO) levels (ng/ml) between the two groups. Values are presented as mean±SEM. There was no significant difference in MPO concentration over time between the two groups

MPO concentration was not significantly different between curcumin and placebo groups at the beginning (0) and 1, 2 and 4 days after the intervention (Figure 1). 

**2. Interleukin 18 (IL-18) concentration**

**Figure 2:**
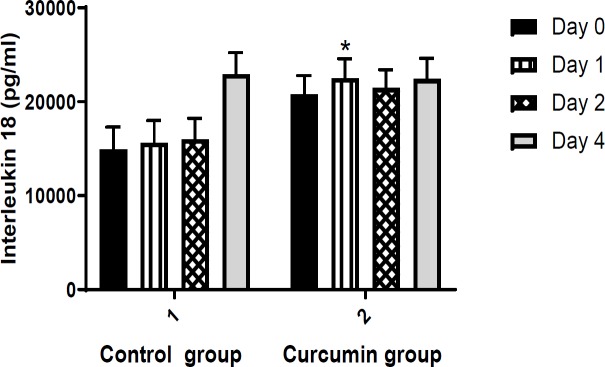
Comparison of interleukin 18 (IL-18) levels (pg/ml) between the study groups. Values are presented as mean±SEM

Serum level of IL-18 was not significantly different between curcumin and placebo groups at the beginning, and 2 and 4 days after intervention. On the first day after the treatment, IL-18 significantly increased in curcumin group in comparison to control group (p =0.043) (Figure 2). 

**3. Matrix metalloproteinase (MMP-9) concentration**

**Figure 3:**
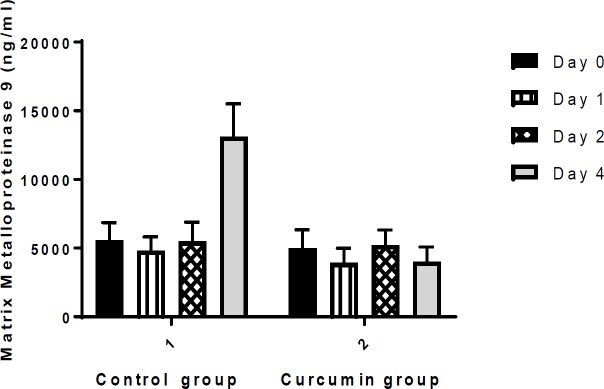
Comparison of matrix metalloproteinase 9 (MMP-9) levels (ng/ml) between the control and curcumin groups. Values are presented as mean±SEM

There was no significant difference in serum levels of MMP-9, between curcumin and placebo group at the beginning, and 1, 2 and 4 days after the intervention (Figure 3).

**Evaluation of relations among serum biomarkers levels and coronary risk factors**

According to the results, none of the risk factors such as gender, smoking or addiction, hypertension, hyperlipidemia, diabetes or age interfered with the levels of evaluated biomarkers. No significant difference in the biomarker levels was observed between high risk and low risk groups. Merely serum level of IL-18 (day 0) was significantly higher in hypertensive and also older groups (p<0.01 and p<0.05, respectively).

## Discussion

This clinical trial study evaluated the effect of curcumin on inflammatory biomarkers in patients with unstable angina. Results showed that administration of nanocurcumin (80 mg/day for five days) to patients with unstable angina could not reduce the level of MPO, IL-18 and MMP-9. It was found that serum level of IL-18 was higher in older people (men over 45 and women over 55 years old) and participants with hypertension. But, other risk factors such as gender, smoking or addiction, hyperlipidemia and diabetes, did not influence blood levels of IL-18, MPO and MMP-9.

Curcumin consumption was limited due to its very low water solubility, which could consequently cause lower bioavailability (Basnet and Skalko-Basnet, 2011 ▶). Thus, new curcumin formulations with higher water solubility and bioavailability, are being considered (Priyadarsini, 2014 ▶; Storka et al., 2015 ▶). It was also revealed that curcumin nanomicelles have higher absorption and its anti-inflammatory property improved for about 22 folds (Anand et al., 2008 ▶). 

Several studies indicated anti-inflammatory properties of curcumin (Anand et al., 2008 ▶). To patients with osteoarthritis, curcumin was administered daily for eight months; curcumin could decrease serum inflammatory biomarkers including IL-1β and IL-6. It was suggested that curcumin can be administered in such patients in combination with nonsteroidal anti-inflammatory drugs (NSAIDs) for a long time, to diminish side effects of NSAIDs (Gupta et al., 2013 ▶). 

Also, the effect of liposomal curcumin (80 mg/day for 4 weeks) on the health of middle- aged participants (i.e. 40-60 years old) was evaluated. This formulation of curcumin decreased amylase in saliva, as well as plasma *triglycerides* (TG), alanine transferase and beta amyloid. Radical scavengers in saliva, nitric oxide and myeloperoxidase were also increased in plasma (Gupta et al., 2013 ▶). 

Some other studies demonstrated that curcumin administration could diminish inflammatory cytokines such as TNF-α, IL-1 or IL-8 (Swarnakar et al., 2005 ▶). Similarly in inflammatory bowel disease (IBD) patients, expression of other biomarkers like IL-1β, IL-10 and MMP-3 was dose-dependently inhibited (Gupta et al., 2013 ▶).

Besides chemotherapy, curcumin was administered to patients with resistant colorectal cancer (0.45-3.6 g/day) for 4 months. No toxic effect was observed at these doses. Curcumin 3.6 g/day led to 57-62% reduction in prostaglandin E2 (PGE2) serum levels 1 hour, 1 day and 29 days after curcumin administration. According to the results, 3.6 g/day curcumin was suggested to be used for prevention of gastrointestinal (GI) cancers (Sharma et al., 2004 ▶).

In people with stable and unstable angina and those with a recent myocardial infarction (MI), IL-18 was significantly higher than the control group. Also, IL-18 concentration was significantly higher in patients with unstable angina compared to patients with stable angina. IL-18 mRNA showed higher expression levels in patients with unstable atherosclerotic plaque (Hulthe et al., 2006 ▶; Rosso et al., 2005 ▶). 

Macrophages play a key role in instability of atherosclerotic plaques by producing MMPs such as MMP-9 (Apple et al., 2005 ▶). Another investigation in peripheral blood mononuclear cells (PBMCs), a reduction in MMP-9 level was observed in rats with inflammation. In fact, delayed reduction in MMP-9 expression occurred as a result of curcumin inhibitory effect on NF-KB and COX (Saja et al., 2007 ▶).

MPO activity is considered a CAD biomarker as it had been significantly elevated in blood in CAD patients. MPO activity was also significantly increased in leukocytes (Apple et al., 2005 ▶). 

In the present study, nanocurcumin administration for 5 days to patients with unstable angina did not affect MPO, IL-18 and MMP-9 levels significantly.

Previous studies demonstrated that curcumin could inhibit MMPs in human lung cancer cells (Lin et al., 2009 ▶). According to these findings, a long-term study should be considered to investigate the effect of curcumin on MMP-9. It was not possible in this study to check the biomarkers for a longer period of time, as the patients with unstable angina were available only for five days. 

It was also proven that the effect of curcumin on MMP is dose-dependent. So, insufficient dose of curcumin administered in this study, may explain the lack of efficacy as well. The effects of higher doses of curcumin on this biomarker should be studied.

Possible inhibitory effects of curcumin on collagen destruction and involved biomarkers such as MPO was investigated. In a clinical study on 22 patients with knee osteoarthritis, the individuals took 6 caps/day of bio-optimized curcumin (42 mg curcumin in each capsule) for 3 months. There were no significant reductions in MPO serum levels following curcumin administration (Henrotin et al., 2014 ▶). Similar results regarding the effect of curcumin on MPO serum concentration in patients with unstable angina were found in the present study.

It is clear that smoking or addiction, hypertension, hyperlipidemia, diabetes and also aging make patients more susceptible to atherosclerosis and plaque instability. So, the influence of risk factors involved in acute coronary syndrome on mentioned biomarkers was investigated. The findings of the present study revealed a direct relation between age (above 45 years for men and 55 years for women) and hypertension, and elevated IL-18 levels.

The main limitation of the present study was the short duration of curcumin administration due to lack of cooperation of patients and problems in obtaining periodic blood samples. Also, according to the above-mentioned problems, the sample size was small which made this investigation a pilot study.

Results showed that administration of curcumin (80 mg/day) to patients with unstable angina was not able to reduce the level of MPO, IL-18 and MMP-9 biomarker. Future studies with larger sample sizes are suggested. It is also recommended to extend the curcumin administration period.

Due to the significant increase in serum levels of IL18 in the patients and the important role of this biomarker in the development of atherosclerosis, IL18 levels should be reduced in patients; also, investigation of IL18 relationship with cardiac complications and cardiac arrhythmias should be examined.
